# Heavy Metal Quantification in Renal Tissue of Patients in the State of Yucatan and Its Association with Urolithiasis

**DOI:** 10.5402/2012/548256

**Published:** 2012-09-27

**Authors:** Luis A. May-Ix, J. Gabriel Rosado-Rubio, Martha Medina-Escobedo, Arturo F. Castellanos-Ruelas, Luis A. Chel-Guerrero, David A. Betancur-Ancona

**Affiliations:** ^1^Faculty of Chemical Engineering, Autonomous University of Yucatan, Campus for Engineering and Mathematical Sciences, Periferico Nte. 33.5 km, Tablaje Catastral 13615, Colonia Hidalgo Chuburna Inn., 97203 Mérida, YUC, Mexico; ^2^Research Laboratory “Agustin O'Horan” Hospital, Health Services of the State Government of Yucatan, 97000 Mérida, YUC, Mexico

## Abstract

A possible cause associated with urinary lithiasis (UL) is the bioaccumulation of heavy metals in the kidney. The aim of this study was to evaluate the content of Cu, Pb, and Cd in kidney tissues removed from patients with nephrological problems and associate it with UL. Samples of 50 kidney sections from patients were analyzed. Results were statistically analyzed using a fixed effects model including the overall mean, the effect of the health status of patients (with or without UL), gender (male and female), the interaction between both factors and the random error (NID  (0, *σ* ^2^)). Cu level was 8.8 ± 4.4 mg/kg (mean ± DS) and 25.5% of samples had levels above normal. Lead content in 97.9% of the samples (3.6 ± 1.5 mg/kg) was above normal. All results of Cd (13.2 ± 16.6 mg/kg) were below the maximum permissible limits. There was no difference in the amount of heavy metals on patients with or without UL (*P* > 0.05) nor depending on the gender (*P* > 0.05). It was concluded that there is no apparent relationship between a very elevated level of Cu or Pb in the kidney on the development of UL.

## 1. Introduction

Urinary lithiasis (UL) is an endemic problem in Yucatan, affecting 5.5% of the population and ranks among the top three pathologies that cause kidney failure, after *diabetes mellitus* and hypertension [1]. This percentage is very high in relation to its prevalence in other states in Mexico, where UL is a low-frequency disease (0.24%) [2, 3].

The deterioration of renal function may be unilateral and in manycases induces nephrectomy. Among the most important causes that induce nephrectomy are hydronephrosis, tumors, and dysplasia. In the United States, a very low percentage of nephrectomy is related to UL [4]. In contrast, in Yucatan, an average of 199 nephrectomies is performed per year, out of which 59.6% are related to UL and to a lesser extent related to tumors, malformations, and other syndromes; 15% of the total cases of nephrectomy are associated with *diabetes mellitus* and/or hypertension [5].

The etiology of urinary stones is multifactorial; among these factors, the most important ones are those associated to eating habits [6], environmental aspects [7], and genetics [8]. As far as eating habits, one predisposing factor to UL is the excessive intake of sodium [9]. As for the environmental aspects, it has been reported that heat is a predisposing factor for UL because of its influence on fluid status and urine volume [10]. Finally, the importance of genetic factors on UL was revealed in a study in Yucatan [11] where UL due to hypocitraturia was the origin of 61.2% of the cases; this paper shows that the GG genotype polymorphism Ala62THr in the ZNF365 gene is associated with hypocitraturia; therefore, metabolic changes associated with polymorphisms in specific genes favor the formation of urinary calculi in the population of Yucatan.

Cattle production systems in Yucatan are very diverse and some of them induce problems of bioaccumulation of contaminants in animal tissues, regardless of quality controls that may be established. Beef carcasses of animals slaughtered in two abattoirs of Merida, the capital city of the state of Yucatan [12], were sampled and it was found that over 78% of renal, muscular, and liver tissue samples had Cu levels above the permitted limits [13] and 67% of the samples had Pb levels higher than what is allowed [13]. Cd was found within normal limits [13]; As and Hg were not detected. Reports on chronic exposure to heavy metals, for example, Pb, results in the bioaccumulation in humans and is associated with cognitive decline in children [14]; it is also associated with hypertension, impaired metabolism of uric acid, *diabetes mellitus*, urinary stones, gout, and other diseases that are all related to UL [15].

Potential exposure to high concentrations of heavy metals in the population of Yucatan through meat consumption could explain, in part, the very high incidence of UL. Therefore, the objective of this study was to determine the content of Cu, Pb, and Cd in kidney tissue obtained from human patients by nephrectomy and establish its relationship with UL and gender.

## 2. Materials and Methods

This experiment was carried out in the city of Merida in the state of Yucatan in Mexico. Renal tissue samples were obtained at the O'Horan Hospital depending on the Yucatan State Health Services and also obtained from the Department of Pathology of the Medical Unit number 25 of the Mexican Social Security Institute, in Yucatan, where most of the nephrectomies are carried out.

### 2.1. Acquisition and Preparation of Samples

This experiment was conducted with the understanding and consent of all subjects involved, as well as a statement written by the Ethical Committee of the Hospital, approving the experiment.

The total number of samples to be taken was determined by a presampling done with seventeen renal tissues, on which Cu was analyzed and the variance (*s* ^2^) of the data was calculated. Variance was incorporated to an equation assuming a *z*-value of 1.96 and an error of ±1.4 mg/kg of Cu [16]. Cu was used because of the ease of its analysis. Based on this calculation, sample size was estimated at 50 renal tissues.

Over a period of six months, samples were collected from patients with various diseases who underwent nephrectomy. Samples were preserved with formalin in polyethylene bags and labeled individually until analysis. Gender, age, and clinical diagnosis of patients were recorded.

Samples were taken from 13 men (26%) and 37 women (74%), and the average age was 42.7 ± 19.9 (mean ± SD years) (minimum 1 year, maximum 75 years).

The analysis of heavy metals, Cu, Pb, and Cd, was carried out in accordance with the Mexican Official Methods of Analysis [17] as follows. Approximately, 2 g of dried samples were weighed in a porcelain crucible of 50 mL; 7.5 mL of magnesium nitrate solution at 6.67% w/v were added to each sample to prevent volatilization of Pb. Samples were thoroughly mixed and allowed to stand for 3 h. Subsequently, the samples were dried at 90–95°C for 4 h. They were placed in an oven gradually raising the temperature, until it reached 300°C. After the cessation of smoke was observed, temperature was increased slowly up to 500–550°C to prevent an abrupt incineration, causing losses of analyte. Temperature was kept for approximately 12 h. After cooling, ashes were completely dissolved with 3 mL of 1 N HCl and placed in polypropylene tubes, the crucibles were rinsed with two additional aliquots of 1 mL of 1 N HCl, then placed in their respective tubes to obtain a volume of 5 mL, finally they were stoppered and mixed thoroughly. A reagent blank was prepared. Subsequently, samples were analyzed by atomic absorption spectrophotometry.

### 2.2. Quantification of Heavy Metals by Atomic Absorption Spectrophotometry

Analyses were carried out using an atomic absorption spectrophotometer (Perkin Elmer, model AAnalyst 800). Working conditions were those described in the instruments operation manual.

The sensitivity of the instrument was verified using standard solutions of known concentration as indicated in the operations manual for each element. Standards used for the preparation of solutions contained 1000 mg/mL of Cu, Pb, or Cd, which were used for the calibration of straight lines in each case. The software provided with the equipment performed the calculation of the linear correlation coefficient (*r*), establishing the linearity at values of *r*≅0.995 or greater. The spectrophotometer was adjusted to zero with the reagent blank. Standard solutions of Cu, Pb, and Cd were introduced, from lowest to highest concentration with at least three replicates, reading the absorbance. After reading the standards (absorbance versus concentration) samples were analyzed.

### 2.3. Statistical Analysis of Data

The data on the content of Cu, Pb, and Cd were transformed into natural logarithms to avoid heteroscedasticity [18]. These variables were statistically analyzed with the least squares method, using a fixed effects model that included the overall mean, the effect of the health status of patients (with or without lithiasis), the effect of gender (male and female), the interaction between both factors, and the random error (NID (0, *σ* ^2^)). Analyses were performed with the SAS statistical package [19] using means and GLM routines, and, finally, correlations between the content of Cu, Pb, and Cd were calculated, and also between the age of the subjects and Pb content.

## 3. Results and Discussion

### 3.1. Average Contents of Heavy Metals

The diagnosis leading to nephrectomy are shown in Table 1. Among the most important diseases associated with nephrectomy are UL in 54% of cases and in a lesser extent, neoplasms, chronic infectious processes, and malformations.

Table 2 shows the content of heavy metals in the kidney samples that were submitted to analysis as well as the comparison with normal levels.

Twenty-five point five percent of samples had a Cu concentration higher than normal. Consumption of beef meat with high Cu content [12] could be contributing in this heavy metal deposition in the kidney of patients. 

The content of Pb in almost all samples was above the threshold considered normal, suggesting that chronic exposure to Pb can represent a risk in UL etiology. Lead is a well-known systemic toxic that can affect the general population. The main sources of exposure are the combustion of gasoline, industrial emissions, consumption of contaminated food (candy in particular), and mouthing or ingestion of contaminated toys. Food and toys increase the possibility of chronic exposure to Pb in both children and adults [15].

The origin of the presence of Pb in human tissues as the last link in the food chain begins with soil contamination. Lead will be absorbed by plants, which are consumed by cattle, and finally passing to man [23]. To confirm this hypothesis in the case of Yucatan, it is necessary to study Pb content in soil and plants from different geographical areas of the State.

Cadmium was found in all cases in normal concentrations. A large variability in the results of Cd was expected, since normal content is up to 120 mg/kg [22]. Previously it was stated that the Cd content in groundwater in the state of Yucatan, was at normal levels [24].

### 3.2. Concentration of Heavy Metals in Patients with or without Lithiasis

Table 3 shows the results on the content of heavy metals in kidney samples depending on the health status of the patients.

There were no statistically differences (*P* > 0.05) in concentrations of Cu, Pb, and Cd in kidney tissues attributed to the patient's illness. Therefore the patients with UL did not have higher kidney PB levels than patients with other kidney pathologies.

### 3.3. Concentration of Heavy Metals by Gender

Table 4 presents the results of the heavy metal content in kidney samples depending on gender.

There were no statistically differences (*P* > 0.05) in concentrations of Cu, Pb, and Cd in kidney attributable to gender.

It is noteworthy that the interaction among variables (presence or absence UL and gender of the patient) was not significant (*P* > 0.05).

### 3.4. Correlation between Variables

A moderate correlation (*P* < 0.01) between Cu content with Pb (*r* = 0.62) and Cd (*r* = 0.58) was found. These results suggest that the high content of all three heavy metals in renal tissues may have a common origin, possibly the consumption of beef with a high content of heavy metals. In the case of Cu and Cd no correlation was found.

Correlation analysis between the age of the subjects with Pb and Cd content of the tissue showed no relationship (*r* = 0.15, *r* = 0.27, resp., *P* > 0.05), suggesting a common source exposure, possible from early stages of life.

These findings are especially interesting, considering that adult hypertension, hyperglycemia, interstitial nephritis and/or renal failure could be a consequence of Pb poisoning [25]. Other clinical situations are also referred: vertebral anomalies, heart defects, renal malformations, and abnormalities on the extremities of newborns of mothers with high Pb in serum during the first trimester of pregnancy [24]. Urinary malformations are the fourth leading cause of health care in the Pediatric Nephrology Unit of the O'Horan Hospital in Yucatan [11], supporting the hypothesis of Pb exposure in neonatal children. Obviously, the metabolic alterations associated with polymorphisms in specific genes, also may induce UL, as discussed above.

Other authors [26] underline the important influences of age, sex, region, and race/ethnicity on UL. The finding of Pb in higher than allowed in meat from cattle suggests the possibility of its influence in the etiology of UL. This is supported by the fact that alterations in the metabolism of uric acid and calcium occur with chronic exposure to Pb [15].

## 4. Conclusions

Elevated levels of Cu and Pb in kidney tissue samples were found. At least 25% of the samples had higher levels of Cu than normal. As to Pb, almost all of the samples (98%) were above normal values. Cd levels were within normal limits. Apparently, this bioaccumulation of heavy metals is not directly associated with UL nor appears to be associated with the gender of individuals.

Although no association could be demonstrated between the contents of Cu, Pb, and Cd in kidney tissue and UL, the findings support the need for further studies to determine the impact specially of lead on population health in Yucatan.

## Figures and Tables

**Table 1:** Clinical diagnosis and indication for nephrectomy of the patients included in the study.

Diagnosis	*n* (%)
Urinary lithiasis	27 (54)
Neoplasia	13 (26)
Chronic infection and atrophy	5 (10)
Malformation	4 (8)
Kidney transplant rejection	1 (2)

**Table 2:** Percentage of kidney samples with higher heavy metal concentration than normal. Mean ± SD.

Heavy metal	Content in kidney tissue (mg/kg)	Normal levels in human kidney (mg/kg)	Percentage of samples above normal
Cu	8.8 ± 4.4	0.70–10.3 [20]	25.5
Pb	3.6 ± 1.5	0.02–0.90 [21]	97.9
Cd	13.2 ± 16.6	0.50–120 [22]	0

**Table 3:** Concentrations of Cu, Pb, and Cd (mg/kg) in kidney depending on the health status of the individual—with or without lithiasis. Mean ± SD.

Heavy metal	Health status		Minimum value		Maximum value	
	With urinary lithiasis (W-UL)	With other kidney pathologies (W-OKP)	W-UL	W-OKP	W-UL	W-OKP
Cu	7.9^a^ ± 4.1	9.6^a^ ± 4.8	2.7	3.8	17.1	20.0
Pb	3.3^a^ ± 1.9	3.8^a^ ± 1.3	0.7	1.4	9.8	6.1
Cd	12.0^a^ ± 16.1	14.5^a^ ± 16.5	0	0.5	63.9	58.2

^a/a^In the same line *P > *0.05.

**Table 4:** Concentrations of Cu, Pb, and Cd (mg/kg) in kidney in relation to gender of the patient (M = male, F = female). Mean ± SD.

Heavy metal	Gender		Minimum value		Maximum value	
	M (*n* = 13)	F (*n* = 37)	M	F	M	F
Cu	10.4^a^ ± 4.4	8.1^a^ ± 4.3	4.5	2.7	17.5	20.0
Pb	3.7^a^ ± 1.3	3.5^a^ ± 1.8	1.4	0.7	5.6	9.8
Cd	17.2^a^ ± 19.7	11.5^a^ ± 14.4	0.6	0	58.2	63.9

^a/a^In the same line *P > *0.05.

## Abbreviations

UL: Urinary lithiasis (kidney stones)
Cu: Copper
Pb: Lead
Cd: Cadmium.
